# The Middle East respiratory syndrome coronavirus in the breath of some infected dromedary camels (*Camelus dromedarius*)

**DOI:** 10.1017/S0950268820002459

**Published:** 2020-10-14

**Authors:** Maged Gomaa Hemida, Mohammed Ali, Mohammed Alhammadi, Abdelmohsen Alnaeem

**Affiliations:** 1 Department of Microbiology, College of Veterinary Medicine, King Faisal University, Al-Hasa, Saudi Arabia; 2 Department of Virology, Faculty of Veterinary Medicine, Kafrelsheikh University, Kafrelsheikh, Egypt; 3 Department of Biological Sciences, College of Science, King Faisal University, Saudi Arabia; 4 Department of Botany and Microbiology, Faculty of Science, Minia University, El-Minia 61519, Egypt; 5 Department of Clinical Sciences, College of Veterinary Medicine, King Faisal University, Al-Hasa, Saudi Arabia

**Keywords:** Breath, dromedary camels, MERS-CoV, phylogenetic analysis, real-time PCR

## Abstract

Dromedary camels remain the currently identified reservoir for the Middle East respiratory syndrome coronavirus (MERS-CoV). The virus is released in the secretions of the infected camels, especially the nasal tract. The virus shedding curve through the nasal secretions was studied. Although human transmission of the virus through the respiratory tract of close contact people with dromedary reported previously, the exact mechanism of transmission is still largely unknown. The main goal of this study was to check the possibility of MERS-CoV shedding in the exhaled air of the infected camels. To achieve this goal, we conducted a follow-up study in one of the dromedary camel herds, December 2018–April 2019. We tested nasal swabs, breath samples from animals within this herd by the real-time PCR. Our results showed that some of the tested nasal swabs and breath were positive from 24 March 2019 until 7 April 2019. The phylogenetic analysis of the obtained S and N gene sequences revealed the detected viruses are clustering together with some human and camel samples from the eastern region, especially from Al-Hufuf city, as well as some samples from Qatar and Jordon. These results are clearly showing the possibility of shedding of the virus in the breath of the infected camels. This could explain, at least in part, the mechanism of transmission of MERS-CoV from animals to humans. This study is confirming the shedding of MERS-CoV in the exhaled air of the infected camels. Further studies are needed for a better understanding of the MERS-CoV.

## Introduction

It has been almost 8 years since the emergence of the Middle East respiratory syndrome coronavirus (MERS-CoV) in 2012 in the Arabian Peninsula. The majority of human cases were reported in the Arabian Peninsula [1–3]. More recently, there are many reports about the potential risk of infection with MERS-CoV in Africa [1]. The only known reservoir so far is the dromedary camels [4]. There are many reports about the animal–human transmission in the context of MERS-CoV [5, 6]. It is now well documented that MERS-CoV is released in the respiratory secretions detected in the nasal swabs by several research groups [5, 7–11]. Although no report on the secretion of the virus in the milk, it is highly recommended to boil it before consumption to avoid any potential contamination of the raw milk during the process of milking and handling [12–14]. Similar concept for eating the meats and raw organs, particularly the liver, it should be cooked well before consumption to avoid any potential hazards of infection as suggested by the World Health Organization (WHO) [12, 14]. The virus was also detected in the air around some positive camel herd. Detection of the MERS-CoV-RNAs of identical sequences from the owner and the herders of this particular herd was also reported [5, 6]. However, some other studies failed to detect the virus in the urine of some infected animals [15]. Earlier studies succeeded in the detection of the MERS-CoV nucleic acids in the air circulated by an infected camel herd as well as in the people of close contact of this particular herd [5, 6]. Meanwhile, several reports showed the possibility of detection of the MERS-CoV nucleic acids in the circulating air in some health care settings during the Korean outbreak of MERS-CoV in 2015 [16, 17]. The main goal of the current study was to check the possibility of MERS-CoV shedding in the infected animal breath.

## Materials and methods

### Animal ethics statements

We conducted this study according to the guidelines of the Animal Ethics protocols and the National Committee of Bio-Ethics, King Abdul-Aziz City of Science and Technology, Royal Decree No. M/59 (http://www.kfsh.med.sa/KFSH_WebSite/usersuploadedfiles%5CNCBE%20Regulations%20ENGLISH.pdf). The animal experiments were approved by the animal care committee of the deanship of scientific research, King Faisal University, Saudi Arabia.

### Dromedary camel herd information

We conducted molecular surveillance for the MERS-CoV in a dromedary camel herd during 2019. This herd consists of 52 animals held together in wire fenced yards. The male animals were kept in a separate individual partition while the female was kept together in multiple partitions. There is a shared open yard where all females may be allowed to be mixed together. The males only approach females during the mating season from November to March per each year.

### Animal selection and samples collection

During the time from 10 March 2019 until 7 April 2019, we randomly selected nine animals out of the 52 (18%) from the camel herd to conduct our molecular surveillance. All animals were of variable ages ranging from 2 to 8 years old. The nine animals selected from different colour coat-based breeds, including Magaheem, Wodoah and Sofr, were selected. These nine animals were mixed with the rest of the animals in the herd all the time. Our molecular surveillance lasts from 10 March to 8 April 2019, with a biweekly sampling interval. We collected nasal swabs, breath samples from these nine animals, as previously described. A paired nasal and breath samples were collected per each animal at each time point, as described in detail below.

### Nasal swabs

Nasal swabs were collected from at least 15% of the dromedary camel herd from November until March 2019. Swabs were transferred into the tubes containing viral transport media including the DMEM media containing 10% foetal bovine sera and antibiotic cocktail (penicillin and streptomycin). The processing of the collected swabs was done as previously described. Briefly, the swabs were vortexed, then centrifuged at 5000 RPM for 10 min in a cooling centrifuge at 4 °C. The supernatants were collected and stored at −80 °C for further testing.

### Breath collection technique

The breath samples were collected in sterile tubes containing viral transport media as used for the collection of the nasal swabs. The breath collection technique was done in a simple way. Briefly, the animals were secured in a specially designed camel crush. The animals were allowed to settle down for 5 min. With the help of an assistant, the nasal orifice is dilated, and the tube containing the media is slightly inserted inside (2–3 cm) the orifice without touching the skin or the mucous membranes of these animals during sampling. The collection tubes were held in a slope position facing the air stream coming from the animal during the exhalation. The tubes were kept in such position until the animal completes at least five cycles of breathing, including five exhalation streams. Any container that touched the skin or the mucous membrane of the animals was discarded and not included in our analysis. The collection tubes were shaken immediately for 3 min and inverted upside down several times, then placed into ice tanks until transferred to the laboratory.

### Viral RNA extraction

The viral RNAs were extracted from paired swabs and breath samples collected from dromedary camels using the Qiagen viral RNA (RNeasy Mini Kit, Qiagen, Hilden, Germany) extraction kit, according to the manufacturer's protocol. The concentration of the extracted viral RNAs was measured by the Nanodrop machine (Thermo Scientific NanoDrop 2000, Applied Biosystems, 850 Lincoln Centre Drive, Foster City, CA 94404, USA). The eluted RNAs were stored at −80 °C for further testing.

### The real-time PCR technique

The RT-PCR targeting the upstream gene (Up-E) of MERS-CoV was used for screening [13]. Confirmation was done using the open reading frame (ORF) 1a. Five μl of the extracted RNA was subjected to the real-time PCR testing using UpE primers using LightMix Molecular Dx MERS-CoV-UpE kits (Roche, Roche Molecular Systems, Inc, Pleasanton, CA 94588, USA) according to the manufacturer's protocol. All positive samples by the UpE assay were confirmed by ORF1a, as previously described [7]. Positive samples were considered when there is an overlapping between the results from two targets.

Samples (nasal swabs and breath) that had Ct values <39 were considered positive per each target.

### Primers and oligonucleotides

We used several sets of primers to amplify the partial MERS-CoV-S, MERS-CoV-N genes. The sequences of these primers are as follow: NF-5′-CCT TCG GTA CAG TGG AGC CA-3′ and NR-5′-GAT GGG GTT GCC AAA CAC AAA C-3′, primers for the MERS-CoV-S gene SF-5′-CCAATTTA-CGCCAGGATGAT-3′ and SR-5′-AATAGAGGCGG AAATAGCAC-3.

### RT-PCR and gel electrophoresis

We used the primers listed above to amplify the partial MERS-CoV-S and N genes using the QIAGEN One-step RT-PCR Kit (Cat No./ID: 210210) to synthesis the cDNA then doing the PCR in one reaction. The procedure of this one-step reaction was carried out as per the kit's instructions as well as previously described. We ran 5 μl per each amplified MERS-N and S amplicons on a 1% agarose gel containing SYBR® Safe DNA Gel Stain (Invitrogen, Thermo Fisher Scientific, Waltham, MA, USA). The amplicons were visualised under ultraviolet light, then the gel pictures were photographed with the gel documentation system (Bio-Rad Laboratories, Inc., Hercules, CA, USA). We purified the desired PCR products from the target bands by using the gel-based PCR extraction kits (Qiagen, Cat No/ID: 28704), as per the instructions of the kits. We eluted the purified PCR products in 50 μl elution buffer as suggested by the kits, then stored at −20 °C.

### Sequencing and sequencing analysis

We sequenced some positive samples from dromedary camels having low Ct values (≤29) on the real-time PCR. The sequencing was done by the Sanger method using the Applied Biosystems^®^ 3500 sequencing machine. We sequenced the purified PCR products in two different directions by the original primers used for amplification above. The obtained PCR products were assembled using the Sequencher 5.4.6 sequencing analysis software (2017 Gene Codes Corporation, Ann Arbor, MI, USA) and performed nucleotide blast in NCBI (https://blast.ncbi.nlm.nih.gov/Blast.cgi?CMD=Web&PAGE_TYPE=BlastHome). These sequences were aligned and compared to other MERS-CoV sequences available in GenBank and the GISAID.

### Phylogenetic analysis

We used the obtained sequences from dromedary camels to develop the phylogenetic tree based on the reported partial MSER-CoV-S and N genes sequences. The phylogenetic trees were built using the neighbour-joining method by the Mega-7 software, as previously described. The scale bar represents the tree distance corresponding to 0.6 nucleotide substitution/nucleotides.

### Statistical analysis

**Fig. 1.**
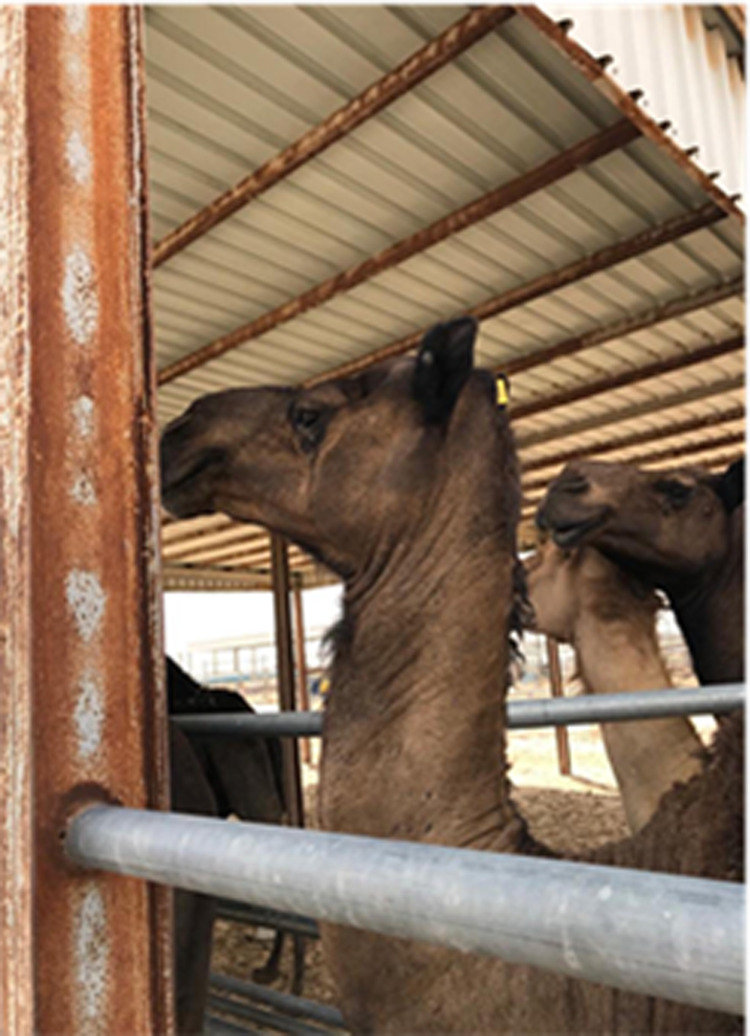
MERS-CoV infection in a Dromedary camels herd. A dromedary camel was showing lacrimation and mild nasal discharge. This animal tested positive by the real-time PCR for MERS-CoV.

A series of two by two tables, utilizing the Fisher's exact test, were used to test the association between the results of the molecular surveillance, the sampling intervals and the sampling technique [18]. All the statistical data analyses were performed by SPSS version 21.0 (IBM Corp, 2012). The values (<0.05) were considered significant (Fig. 1) .

## Results

### Molecular testing of dromedary camel herd for the MERS-CoV nucleic acids in the nasal swabs and breath

Our molecular testing for the selected animals at three time points starting 10 March 2019, until 7 April revealed that 1/9 (11%) of the tested nasal swabs were positive; all the nine animals were negative from the breath samples (Table 1). Two weeks later, on 24 March, 6/9 (77%) nasal swabs were positive, and 5/9 (55%) breath samples were positive (Table 1). Finally, on 7 April, 3/9 nasal swabs (33%) were positive, while 1/9 (11%) of the breath samples were positive (Table 1). The prevalence of positive nasal and breath samples was higher on the second sampling batch (24 March) compared to first (10 March) and third (7 April) batches. However, the difference was statistically significant (*P* < 0.05) only between the first *vs.* second sampling batches. Similarly, consolidated results obtained from both nasal swaps and breath were significantly higher in the second batch *vs.* the first batch (*P* < 0.05). No significant statistical differences were observed between the prevalence of positive results obtained from nasal swabs and breath samples. The detection of the MERS-CoV-RNAs in the breath of all three time intervals was statistically significant (*P* > 0.05) (Table 1).

**Table 1.** Results of the real-time PCR testing for the MERS-CoV in the nasal and breath samples – 2019

Batch/type of samples	1st batch 10 March 2019	2nd batch 24 March 2019	3rd batch 7 April 2019
Nasal	1/9^a^	6/9^a^	3/9
Breath	0/9^b^	5/9^b^	1/9
Total	1/18^c^	11/18^c^^,^^d^	4/18^d^

a *P* < 0.05 for 1st batch nasal samples *vs*. 2nd batch.

b *P* < 0.05 for 1st batch breath samples *vs*. 2nd batch.

c *P* < 0.05 for the sum of nasal and breath samples from 1st batch *vs*. 2nd batch.

d *P* < 0.05 for the sum of nasal and breath samples from the 2nd batch *vs*. the 3rd batch; comparisons were performed by Fisher's exact test.

### Molecular characterisation and phylogenetic analysis of MERS-CoV circulated in the dromedary camel herd 2019

**Fig. 2.**
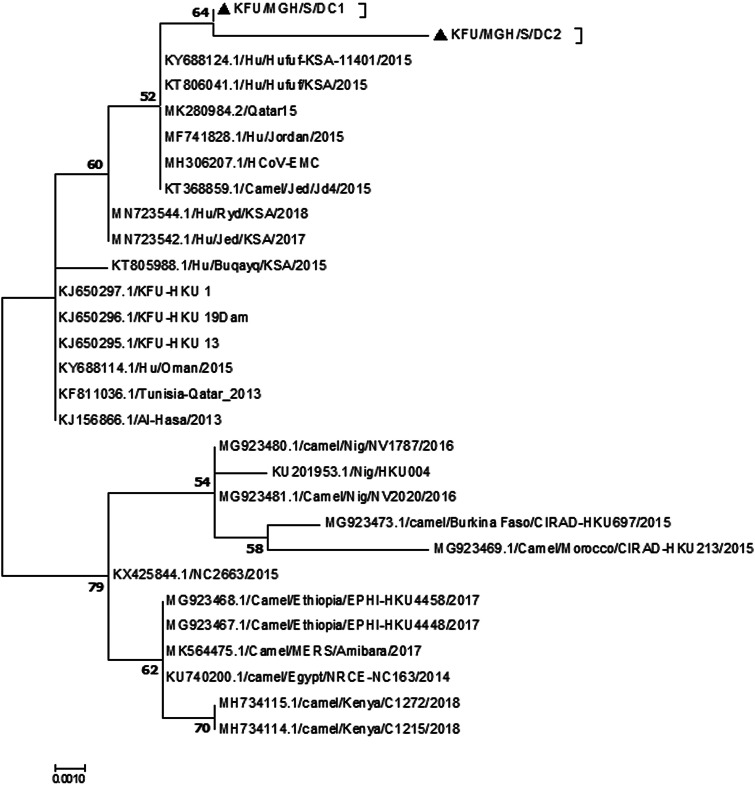
Phylogenetic analysis based on the partial MERS-CoV-S gene. Phylogenetic analysis based on the partial MERS-CoV-S gene from doves and dromedary camels shared the habitat. The maximum likelihood based on the partial MERS-CoV-S sequences revealed these sequences clustered together and share a high degree of identity with other sequences reported from eastern Saudi Arabia, Qatar and Jordon reported in 2015. Our sequences are identified with black triangles.

**Fig. 3.**
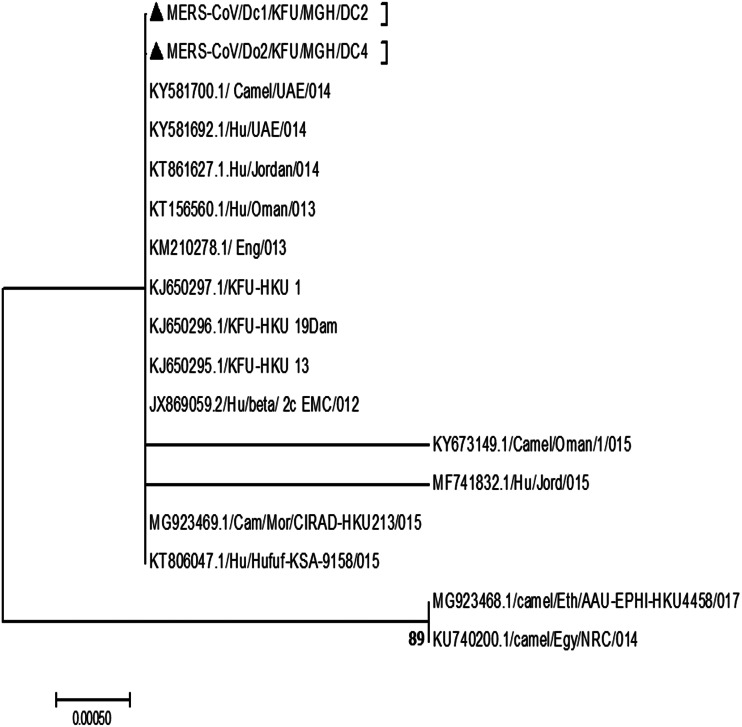
Phylogenetic analysis based on the partial MERS-CoV-N gene. Phylogenetic analysis is based on the partial MERS-CoV-N gene from dromedary camels sharing the habitat. The maximum likelihood based on the partial MERS-CoV-S sequences revealed these sequences clustered together and share a high degree of identity with other sequences reported from eastern Saudi Arabia, Qatar and Jordon reported in 2015. Our sequences are identified with black triangles.

The sequencing analysis of the partial MER-CoV-S and N genes (Figs 2 and 3, respectively) from this positive MERS-CoV herd showing the detected viruses were closely related to the sequence from dromedary camels and humans from the Arabian Peninsula. The reported partial MERS-CoV-S gene clustered together with the other human sequences reported from Al-Hufuf city in the same regions in 2015 as well as sequences from Qatar and Jordon-2015 (Fig. 2).

## Discussion

The MERS-CoV continues to pose a significant risk for humans, especially some of those who may come in close contact with dromedary camels [12, 13]. Despite the emergence of SARS-CoV-2 late 2019 [19], MERS-CoV still has the high case fatality rates among the affected populations compared to SARS-CoV and SARS-CoV-2. The MERS-CoV case fatality rate started at 52% in 2012 then dropped down to 32% in 2019 [20]. The presence of MERS-CoV in the air of the proximity of patients and infected dromedary camels was previously documented independently by many research groups [6, 16, 17]. This result confirms that MERS-CoV may be transmitted through droplet infection. However, little is still known about the mechanism of transmission of MERS-CoV from the dromedary camels to humans. The roles of some camel secretions and excretions in the context of the MERS-CoV infection have been studied [5, 13, 15]. Our results show that about 11% of the tested nasal swabs were positive during the first batch of the collection, while none of the breath samples was positive (Table 1). The reasons behind the inability to detect the viral RNAs in the breath during the first round of sampling may be attributed to the dilution of the viral RNA in the breath, and the RNA may be present at the non-detectable limit by the real-time PCR. A high consistency was observed between the detection of the viral nucleic acids in both the nasal and breath samples in all three tested batches (*P* > 0.05), indicating similar opportunities to detect the virus nucleic acid in both methods (Table 1). This may be attributed to several factors. First, during the early stage of the virus infection, the RNA concentration in the breath may be at an undetectable level by the real-time PCR. Second, the virus replication reached the peak of each animal; therefore, high virus concentration was achieved as previously reported [7, 8]. These results showed that MERS-CoV could be shed in the breath of infected animals for a while. However, the nasal swabs are still the sample of choice in the diagnosis of MERS-CoV among the infected dromedary camel population. Spreading of the virus from animal to another in the same herd could potentially happen through the respiratory routes taking into consideration the very close contact between animals within the same herd. It may also occur through sharing the contaminated food and water from infected animals; however, all these aspects still need further investigations. Detection of the virus in the air of positive camel's herd [5, 6] may suggest the virus is excreted in the breath of the infected animals in high concentration. However, this hypothesis was never tested before. Detection of the foot and mouth diseases virus (FMDV) in the breath of some infected cattle was previously documented [21]. This study confirmed the potential spread of the FMDV between animals and among various herds in close proximity or within a distance. The aim of our study was to test the possibility of MERS-CoV shedding in the breath of the infected dromedary camels. Our results are clearly showing the potential secretion of MERS-CoV in the breath of infected animals (Table 1). The phylogenetic analysis based on the partial MERS-CoV-S and N genes in these infected animals revealed high identity to other MERS-CoV previously detected in the Arabian Peninsula (Figs 2 and 3) [7, 8, 11]. A recent study showed that the SARS-CoV-2 could be transmitted through the droplet infection in the air [22].

**Fig. 4.**
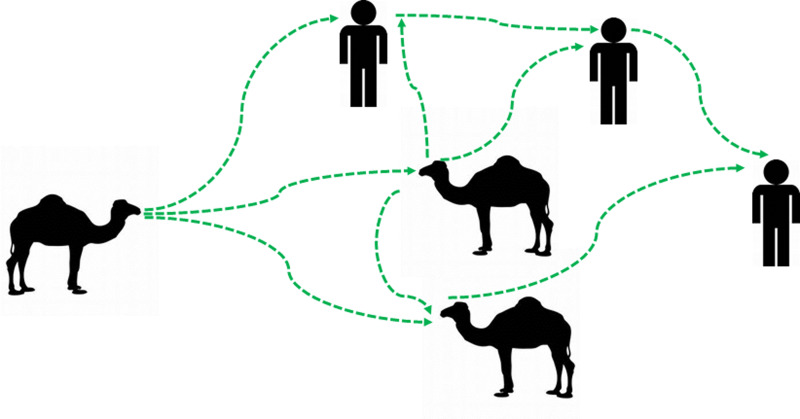
Updated potential hypotheses for the camel/camel and camel/humans interactions in the context of MERS-CoV transmission cycle. An illustration is showing the potential updated transmission hypotheses of MERS-CoV between animals and between animals and humans. The MERS-CoV-infected dromedary camels can transmit the virus to other animals in their proximity through the breath. The virus spreads between animals among specific animal populations. These infected animals may act as a source of infection to humans who came in their close contacts. The virus can be transmitted from humans to humans. Green arrows indicated the path of transmission of MERS-CoV infection between animals and humans.

Although detection of the MERS-CoV-RNAs in the breath does not conclude the viability of the detected virus particles. This may require virus isolation and the plaque assay to ensure the virus infectivity in the collected samples. These techniques should be done at a biosafety contaminant-3 laboratory. However, the overlapping between the viral detection in the nasal swabs and breath, as one of the gold standard techniques for the detection of the virus as well as the high degree of identity of the reported sequences in this study, may provide a piece of strong evidence about the potential shedding of the MERS-CoV in the breath of infected animals at various levels. Further large-scale studies are highly recommended to document the curve of the MERS-CoV shedding in various body secretions and execrations as well as in the breath. This will lead to a better understanding of the dynamics of the virus spread among certain dromedary camel populations as well as in the environment. Taken together all these evidence, we may conclude that MERS-CoV could be transmitted through the breath of infected animals. The virus spread from animal to animal and from animal to human come in their close contact (Fig. 4). However, large-scale controlled studies are still required to further enrich our understandings about the mechanism of MERS-CoV transmission between animals as well as from animals to humans.

## Data Availability

Data are available upon request.
